# Event and Comment

**Published:** 1910-02-15

**Authors:** 


### DENTAL REGISTER.

**Vol. LXIV. February 15, 1910. No. 2.**

### EVENT AND COMMENT.

### PUBLIC MINISTRATION TO THE POOR CHILDREN.

We present in this issue a symposium of the best we can find on the subject of public ministration to the poor children in various parts of our country, who can not have the services of the dentist to repair and prevent diseases of the teeth. This is at this time a most popular and fascinating topic and in many parts of our country the work is being organized and carried out in a most worthy and promising manner. The work in most places has assumed such large proportions that its advocates are greatly embarassed in their efforts to properly care for all the children presenting for treatment. In the cities of New York and Boston hospitals and charitable organizations are freely offering their facilities for the work, and now the sad fact that should have been seen in the first place has become known, that there is an insufficient force of dentists to cope with the problem. It is not unlike the bargain day rush at the department store, except that it is always possible to hire enough clerks to care for such an avalanche of people, who want much for little. In this case we are wholly unprepared to meet a condition that has existed under our very noses, and we would not see that people have wanted to have their teeth cared for, but have not had the knowledge or opportunity to secure this most valued service. One of the essayists makes the statement that there are not enough dentists in the United States to care for the teeth of the people of New York City alone. The best statistics available show us that not more than ten percent of our people have anything like efficient dentistry, in fact not over five percent have adequate service. The problem they are now experiencing in New York and Boston is a famine of dentists, and there seems at the present time no relief in sight. The tremendous advances made in our profession in the last five years are not appreciated by the profession en masse. The exacting requirements brought on by modern methods of orthodontic treatment and oral prophylaxis are taxing our conscientious technical men to their utmost to bring their rescue methods up to our present ideals. Slovenly dentistry is no longer tolerable. These new ideals are calling on us for all the available qualified men, and there are none to spare for benevolent work which offers no remuneration save the rewards of benevolence and charity. In these days of expensive living we are not likely to turn many capable operators into the charity wards of free dispensaries or hospitals.

Personally we welcome this propaganda and its astounding results, not that we intend to plunge headlong into its solution; but because it looks like a great awakener to a realization of the fact that there is now an insistent demand for a more rational treatment of dental diseases. We have spent entirely too much effort and time on tedious and painful reparative or technical procedures. We must meet this problem with a more scientific solution. To us this means that our future study must be for preventive measures that can be administered with less labor and expense. We must find a remedy for dental caries and peridental diseases. This means, investigation by scientists, which we now haven't but the people will soon demand.

